# A novel approach in the identification of microRNAs in malignant pleural effusion for lung cancer diagnosis

**DOI:** 10.3389/or.2025.1642661

**Published:** 2026-01-21

**Authors:** Jesús Valencia-Cervantes, Gustavo Ramirez-Martínez, Margarita Isabel Palacios-Arreola, Alejandra Loaeza-Román, Martha Patricia Sierra-Vargas

**Affiliations:** 1 Departamento de Investigación en Toxicología y Medicina Ambiental, Instituto Nacional de Enfermedades Respiratorias Ismael Cosío Villegas (INER), Mexico City, Mexico; 2 Estancias Posdoctorales por México 2022 (1), Secretaría de Ciencia, Humanidades, Tecnología e Innovación (SECIHTI), Mexico City, Mexico; 3 Laboratorio de Inmunobiología y Genética, Instituto Nacional de Enfermedades Respiratorias Ismael Cosío Villegas (INER), Mexico City, Mexico

**Keywords:** cancer biomarkers, lung adenocarcinoma, squamous cell lung cancer, malignant pleural effusion, MicroRNAs

## Abstract

**Introduction:**

Pleural effusion, an atypical accumulation of fluid in the pleural space, has been identified as a potential indicator of several diseases, including lung cancer. The presence of biomarkers in malignant pleural effusion has been a subject of investigation; however, the expression of microRNAs has received limited attention. The objective of this study is to present a narrative review of the current scientific literature regarding the presence of microRNAs in malignant pleural effusion and their association as new biomarkers in the diagnosis of lung cancer.

**Method:**

A comprehensive search was conducted using the databases: PubMed, ScienceDirect, and EBSCO to identify all original scientific articles published through 30 April 2025. The following terms were utilized in the search: “MicroRNA AND pleural effusion AND lung cancer”, “microRNA AND pleural effusion AND lung adenocarcinoma”, “microRNA AND pleural effusion AND lung squamous cell carcinoma”, “miRNA AND pleural effusion”, miRNA AND pleural effusion AND lung cancer”, “miRNA AND pleural effusion AND lung adenocarcinoma”, “miRNA AND pleural effusion AND lung squamous cell carcinoma”.

**Results:**

A total of 17 studies were identified that distinguished between 106 microRNAs. These studies demonstrated the most significant overexpression and downexpression in lung cancer patients compared to patients without malignancy. However, eight of these studies distinguished between 17 microRNAs expressions and exhibited elevated area under the curve values, sensitivity, and specificity for the involvement in several hallmarks of lung cancer.

**Conclusion:**

The regulatory mechanisms governing microRNAs in malignant pleural effusion are intricate and involve multiple genes that play pivotal roles in several cancer mechanisms. These mechanisms encompass but are not limited to, processes such as cell growth, migration, drug resistance, proliferation, apoptosis, invasion, angiogenesis, and apoptosis.

## 1 Introduction

Pleural effusion (PE) is an atypical accumulation of fluid within the pleural space. A minimal amount of fluid is continuously produced and absorbed in this space to ensure its lubrication and facilitate unobstructed lung movement during respiration. However, several diseases have the potential to disrupt this balance, resulting in an excessive accumulation. The etiology of PE comprises two Light’s criteria, “transudate” and “exudate.” A transudate is an alteration in hydrostatic or oncotic pressures within the pleural space arising from various conditions, including congestive heart failure, nephrotic syndrome, liver cirrhosis, hypoalbuminemia, or peritoneal dialysis. Conversely, the exudates are driven by a variety of factors, including, but not limited to, pneumonia, tuberculosis, malignancy, inflammatory disorders, pancreatitis, lupus, rheumatoid arthritis, and drugs such as methotrexate, amiodarone, phenytoin, and dasatinib (1).

The etiology of malignant pleural disease (MPD) can be classified into three origins: direct extension of an adjacent tumor, pleural metastases of distant tumors, or a primary pleural tumor. The manifestation of MPD can take the form of either solid disease or malignant pleural effusion (MPE), associated with a high degree of morbidity and mortality. The presence of MPE has been associated with a decline in quality of life and the manifestation of debilitating symptoms, including dyspnea, cough, and pain (2). The most common etiologies associated with direct, adjacent, or hematogenous MPE involvement include lung cancer (LC), breast cancer, and hematologic malignancies (3).

In 2019, the World Health Organization projected that it would be among the top two causes of death worldwide before the age of 70 years, with nearly 19.3 million new cases expected, while the death rate reached 10 million (4). More than 85% of cases are classified as non-small-cell lung cancer (NSCLC), with a 5-year survival rate. The two predominant histological phenotypes are lung adenocarcinoma (LUAD, ∼50%) and lung squamous cell carcinoma (LSC, ∼35%). In general, LUAD arises in more distal airways, whereas LSC arises in proximal airways and is more strongly associated with smoking and chronic inflammation than LUAD (5). The presence of MPE is frequently suggestive of advanced-stage malignancy, with overall survival (OS) rates ranging from three to 12 months following initial diagnosis (6).

Early detection of LC is essential to achieve a substantial reduction in cancer-related morbidity and mortality. Therefore, it is imperative to identify novel biomarkers capable of predicting outcomes and tumor response to optimize the therapeutic efficacy. A comparative analysis of diagnostic procedures reveals that pleural cytology and pleural biopsy tests are characterized by minimal invasiveness, provide objective results, and are relatively inexpensive. Reliable and non-invasive testing facilitates early specialist referral, minimizes diagnostic delays, and optimizes access to clinical trials. Several biomarkers have been evaluated for their diagnostic accuracy in MPE, including epigenetic markers such as microRNAs (miRNAs) (7–10).

MiRNAs are a family of noncoding small molecules that range in size from 21 to 25 nucleotides. The molecular mechanism of action of these molecules is characterized by their binding to complementary sites on target messenger RNA (mRNA), thereby inducing either the inhibition of translation or the promotion of mRNA degradation. The post-transcriptional control and subsequent degradation of target mRNA by miRNAs are implicated in various biological processes, including development, body patterning, stem cell differentiation, and tissue identity (11). Non-invasive methods are available for the collection of blood and other bodily fluids, including stool, urine, and saliva. This methodological framework enables longitudinal measurement of miRNAs in a variety of diseases, thereby facilitating disease monitoring and research (12, 13). The extensive regulatory role of miRNAs in all stages of tumor development, coupled with their tissue-specific expression in many cases, underscores their potential as diagnostic and prognostic tools. Aberrant expression of these molecules in cancer cells has been demonstrated to facilitate the maintenance of proliferative signaling, evasion of growth suppressors, resistance to cell death, activation of invasion and metastasis, and induction of angiogenesis (14–16). The presence of cell-free or circulating miRNAs has been detected in several biological samples, including blood, serum, plasma, and tissues from patients diagnosed with LC. This finding suggests that the presence of miRNAs may have significant clinical implications (17–20). Indeed, a number of biomarkers have been evaluated, and their diagnostic accuracy for MPE has been assessed in a growing number of studies. In this study, we present a narrative review of the current scientific literature regarding the identification of miRNAs in MPE as biomarkers in the diagnosis of LC.

## 2 Information search

A comprehensive literature review was conducted using PubMed, Science Direct, and EBSCO to identify all original scientific articles published through 30 April 2025. The search terms employed included “microRNA AND pleural effusion AND lung cancer,” “microRNA AND pleural effusion AND lung adenocarcinoma,” and “microRNA AND pleural effusion AND lung squamous cell carcinoma”, miRNA AND pleural effusion”, miRNA AND pleural effusion AND lung cancer”, miRNA AND pleural effusion AND lung adenocarcinoma”, “miRNA AND pleural effusion AND lung squamous cell carcinoma”. A thorough delineation of the search strategy is provided in Table 1.

**TABLE 1:** Summary of the search strategy.

Items	Specification
Date of search	• 30 April 2025
Databases	• PubMed, ScienceDirect and EBSCO
Search terms used	• The following terms were utilized in the search: “MicroRNA AND pleural effusion AND lung cancer”, “microRNA AND pleural effusion AND lung adenocarcinoma”, “microRNA AND pleural effusion AND lung squamous cell carcinoma”, “miRNA AND pleural effusion”, miRNA AND pleural effusion AND lung cancer”, “miRNA AND pleural effusion AND lung adenocarcinoma”, “miRNA AND pleural effusion AND lung squamous cell carcinoma”
Timeframe	• Manuscripts published after 2010 to 30 April 2025
Inclusion and exclusion criteria	Inclusion criteria • The research manuscript conducted on human pleural effusion (PE) • Including patients diagnosed with lung adenocarcinoma (LUAD), lung squamous cell carcinoma (LSC), or lung mesothelioma (LUM) • The results of the study indicate the fold change, sensitivity, and specificity of malignant pleural effusion of lung cancer: lung adenocarcinoma (MPELUAD), malignant pleural effusion of lung squamous cell carcinoma (MLSC), malignant pleural effusion of lung mesothelioma (MPELUM), as well as benign pleural effusion (BPLE) Exclusion criteria • The study was not a clinical trial, meta-analysis, or review • Studies conducted in animal models were not included in this analysis • To ensure the integrity of the research, studies that did not report the fold change, sensitivity, or specificity were not included in the analysis
Selection process	• Manuscripts were selected independently by the authors using a search strategy to identify relevant documents • Manuscripts detected through this process were then screened for the presence of additional references • A comprehensive search of relevant bibliographic databases yielded a total of 179 results in PubMed, 154 results in ScienceDirect, and 14 results in EBSCO. Following a thorough analysis of the available data, a total of 17 studies were identified that distinguished between 106 miRNAs
Any additional considerations	• The present review was expanded by the incorporation of additional manuscripts, which were included with the objective of enhancing comprehensibility

## 3 Discussion

A total of 17 studies were recognized that distinguished 106 miRNAs. The present review elucidates the most significant overexpression and downexpression in MPE with LC patients and controls, non-malignant subjects, as illustrated in Table 2. However, in eight of these studies, a distinction of 17 miRNAs, including miR-19a, miR-19b, miR-21, miR-22, miR-24, miR-25, miR-29c, miR-30d, miR-126, miR-134, miR-1-3p, miR-144-5p, miR-145, miR-150-5p, miR-182, miR-185, and miR-198. As shown in Table 3, the eight studies exhibited elevated AUC values, sensitivity, and specificity of these miRNAs that participate in LC progression.

**TABLE 2:** Differential miRNAs expression in MPE of patients with LC compared to BPE.

Author	Country	Cases (n)	Male: Female	MiRNA	MPE	BPLE	Fold change	q value
Han et al., 2013 (21)	Korea	MPELUAD (45) BPLE (42)	23:22 29:13	miR-198	​	​	2.10	0.002
				miR-185			1.74	0.114
				miR-134			1.54	0.007
				miR-22			1.49	0.030
				miR-153			1.39	0.091
				miR-296-3p			1.26	0.001
				miR-197			1.21	0.007
Shin et al., 2014 (22)	Korea	MPELUAD (45) BPLE (42)	23:22 29:13	miR-134	4.20	19.75	16.99	0.05
				miR-185	0.008	1.22	0.092	0.05
				miR-22	0.008	1.07	0.051	0.05
Ak et al., 2015 (23)	Turkey	MPELUM (18) BPLE (6)	9:9 5:1	miR-484	7.06	9.54	5.58	0.010
				miR-320	6.62	8.14	2.87	0.017
				let-7a	8.85	12.65	13.93	0.019
				miR-744	11.67	13.76	4.26	0.019
				miR-20a	10.65	13.16	5.7	0.019
				miR-193b	8.09	9.69	3.03	0.019
				let-7d	10.18	12.72	5.82	0.045
				miR-125a-5p	8.8	11.83	8.17	0.045
				miR-92a	9.93	11.19	2.39	0.045
				miR-155	8.77	10.43	3.16	0.045
				miR-152	11.93	13.48	2.93	0.047
Cappellesso et al., 2016 (24)	Italy	MPELUM (29) RMC (24)	17:12 14:10	miR-19a	​	​	2.64	0.05
				miR-19b			0.03	0.05
				miR-21			20.87	0.001
				miR-126			0.12	0.01
Wang et al., 2017 (25)	China	MPELUAD (6) BPLE (2)	4:2 1:1	miR-205-5p	225.24	0.00	21.10	0.05
				miR-483-5p	102.86	0.00	19.97	0.05
				miR-375	625.99	0.85	9.51	0.05
				miR-200c-3p	2058.80	9.14	7.81	0.05
				miR-429	239.93	7.46	5.00	0.05
				miR-200b-3p	2612.72	94.23	4.79	0.05
				miR-200a-3p	1411.23	43.14	5.03	0.05
				miR-203a-3p	365.65	15.73	4.53	0.05
				miR-141-3p	581.27	17.14	5.08	0.05
				miR-148a-3p	4974.37	12624.27	1.34	0.05
				miR-451a	450.51	798.54	−0.82	>0.05
				miR-150-5p	2731.76	1240.25	1.13	>0.05
Hydbring et al., 2018 (26)	Sweden	MPELUAD (18) BPLE (18)	8:10 15:3	miR-375	​	​	57.21	1.06E-06
				miR-200c			194.69	1.31E-05
				miR-141			67.52	1.41E-05
				miR-200b			22.24	6.62E-06
				miR-200a			4.63	1.04E-05
				miR-889			0.44	2.33E-05
				miR-143			0.21	4.78E-05
				miR-145			0.13	4.25E-05
				miR-199a-3p			0.16	7.46E-05
				miR-203			11.35	2.04E-04
				miR-224			2.51	1.13E-03
				miR-301			2.41	9.07E-04
				miR-135a			2.30	1.08E-03
				miR-449			1.73	9.85E-04
				miR-93			2.05	1.28E-03
				miR-885-5p			0.52	1.31E-03
				miR-135b			5.33	1.45E-03
Liu et al., 2018 (27)	China	MPELUAD (9)	​	miR-21	69.14	15.04	​	˂0.0001
		MPELUM (1)		miR-24	73.35	16.32		0.0054
		BPLE (20)		​	​	​		​
Tamiya et al., 2018 (28)	Japan	MPELUAD (41) BPLE (15)	28:13 8:7	miR-21	​	​	15.53	0.001
				miR-31			0.02	0.001
				miR-182			0.0001	0.001
				miR-210			0.05	0.001
Birnie et al., 2019 (29)	Australia	MPELUM (26) BPLE (10)	23:3 7:3	miR-200b	​	​	0.004	0.023
				miR-200c			0.013	0.01
				miR-143			0.020	0.026
				miR-200a			0.030	0.034
				miR-203			0.074	0.032
				miR-31			0.298	0.012
				miR-874			0.4818	0.023
				miR-944			5.700	0.2020
				miR-139-5p			3.418	0.0057
				miR-210			2.590	0.0052
				miR-320			2.380	0.0220
Yu et al., 2019 (30)	China	MPELUAD (25) BPLE (30)	​	miR-1269a	​	​	9.48	0.0002
				miR-205-5p			8.24	0.0001
				miR-429			8.018	0.00009
				miR-196a-3p			8.0007	0.002
				miR-200a-5p			7.85	0.0001
				miR-615-3p			7.57	0.0008
				miR-493-5p			7.46	0.004
				miR-18b-5p			4.28	0.04
				miR-4772-3p			2.68	0.04
Roman-Canal et al., 2019 (31)	España	MPELUAD (9) BPLE (20)	​	miR-150-5p	​	​	3.65	1.87E-03
				miR-144-5p			−11.37	3.76E-04
				miR-1-3p			−13.78	1.41E-06
				miR-584-5p			−9.55	1.90E-08
				miR-133b			−7.72	1.30E-03
				miR-451a			−3.29	3.50E-04
				miR-27a-5p			4.93	8.05E-05
				miR-21-3p			5.65	1.99E-04
				miR-199a-5p			−7.73	3.47E-04
				miR-1249-3p			6.70	5.62E-04
				miR-485-3p			4.80	1.82E-03
				miR-20b-5p			−8.18	3.05E-04
				miR-181c-5p			−3.79	2.06E-03
				miR-30e-5p			−6.46	1.45E-03
Bao et al., 2020 (32)	China	MPELUAD (24) BPLE (41)	​	miR-195-5p	1.10	0.35	3.14	<0.05
				miR-182-5p	1.24	0.76	2.75	<0.05
				miR-34a-5p	0.79	0.35	2.25	<0.05
Shojaee et al., 2022 (33)	USA	MPELUAD (8)	​	miR-1246	8.51	0.22	​	0.021
		BPLE (8)		miR-150-5p	6.32	2.79		0.879
Zhu et al., 2022 (34)	China	MPELUAD (64)	​	miR-21	∼6.5	∼2.5	​	0.05
		BPLE (112)		miR-29c	0.00	∼2.5		0.05
		​		miR-182	∼4.0	∼1.9		0.05
Sun et al., 2024 (35)	China	MPELUAD (12) BPLE (12)	8:4 8:4	miR-6721-5p	∼7.5	∼0.01	​	0.01
				miR-10527-5p	∼5.5	∼0.2		0.001
				miR-4433a-3p	∼10.0	∼1.0		0.001
				miR-3184-3p	∼8.0	∼0.01		0.01
				miR-181a-2-3p	∼18.5	∼1.5		0.001
Chee et al., 2025 (36)	Australia	MPELUM (11) MPELUAD (11) BPLE (7)	10:1 4:7 7:0	miR-145-5p	​	​	1.38	0.03
				miR-1246			4.75	0.02
				miR-141-3p			1.63	0.02
				miR-200-3p			2.86	<0.001
				miR-9-5p			1.78	0.02

**TABLE 3:** Area under the curve (AUC), sensitivity and specificity of miRNAs in the MPE of patients with LC.

Author	MiRNA	AUC	95% CI	Sensitivity (%)	Specificity (%)
Xie et al., 2011 (37)	miR-24	0.71	0.57–0.82	86.0	52.9
	miR-30d	0.75	0.62–0.85	55.8	88.2
Han et al., 2013 (21)	miR-198	0.88	0.80–0.94	71.1	95.2
Shin et al., 2014 (22)	miR-134	0.72	0.61–0.81	80.0	57.1
	miR-185	0.88	0.79–0.94	77.8	90.5
	miR-22	0.83	0.73–0.90	68.9	85.7
Cappellesso et al., 2016 (24)	miR-19a	0.69	0.54–0.83	88.0	52.0
	miR-19b	0.68	0.54–0.83	42.0	90.0
	miR-21	0.79	0.66–0.91	100	45.0
	miR-25	0.59	0.43–0.75	54.0	72.0
	miR-126	0.71	0.57–0.85	83.0	52.0
Liu et al., 2018 (27)	miR-21	0.87	0.73–0.95	80.0	95.0
	miR-24	0.86	0.71–0.94	80.0	95.0
Roman-Canal et al., 2019 (31)	miR-1-3p	0.92	0.93–0.94	93.8	94.3
	miR-144-5p	0.92	0.87–0.88	77.6	94.1
	miR-150-5p	0.93	0.81–0.83	66.6	92.5
Huang, 2020 (38)	miR-145	0.77	0.68–0.86	64.7	79.6
Zhu et al., 2022 (34)	miR-21	0.80	0.74–0.86	73.2	79.3
	miR-29c	0.79	0.73–0.85	72.4	78.2
	miR-182	0.84	0.79–0.89	75.6	80.5

As presented in Table 4, the miRNA families detected in MPE from LC patients are enumerated, along with the total number of predicted human miRNA targets obtained from the MicroRNA Target Prediction Database (https://mirdb.org/). The upregulation and downregulation of miRNAs represent a multifaceted interaction with multiple genes that play a pivotal role in various cancer mechanisms, including cell growth, migration, intracellular lipid content, drug resistance, proliferation, apoptosis, invasion, and angiogenesis. The subsequent section provides a comprehensive description of the cellular mechanisms of miRNAs recognized in LC.

**TABLE 4:** MiRNAs families and isotypes recognized in MPE from LC patients. In addition, the total number of predicted human miRNA targets using the MicroRNA Target Prediction Database (https://mirdb.org/).

MiRNA family	MiRNA isotype	Total target prediction	Gene regulation by miRNAs
miR-1	miR-1-3p	945	PRC1 (40); c-Met, HGF (41)
	miR-1-5p	465	
miR-19	miR-19a-3p	1328	MXD1 (45); MTUS1 (46)
	miR-19a-5p	756	
	miR-19b-1-5p	755	
	miR-19b-2-5p	755	
	miR-19b-3p	1329	
miR-21	miR-21-3p	595	EGFR (48); PPARGC1B (50)
	miR-21-5p	469	
miR-22	miR-22-3p	509	SNAI1, TGFβ1, E-cadherin, Snail, N-cadherin (54); IL1B, NFKB1, SIRT1, FGFR1 (55); c-MET, STAT3, PARP (56)
	miR-22-5p	655	
miR-24	miR-24-1-5p	213	MMP9, WWOX (59); ZNF367 (60)
	miR-24-2-5p	214	
	miR-24-3p	959	
miR-25	miR-25-3p	919	EGFR (62); MOAP1 (63); KLF4, vimentin, MMP11, N-cadherin, E-cadherin (64); CDH1 (65); LATS2 (67)
	miR-25-5p	51	
miR-29	miR-29a-3p	1035	ITGB1, MMP2 (71); Sp1, TGFβ1, TTF-1, E-cadherin, vimentin (73); VEGF (74,77); Bcl-2, Mcl-1 (75)
	miR-29a-5p	885	
	miR-29b-1-5p	695	
	miR-29b-2-5p	859	
	miR-29b-3p	1034	
	miR-29c-3p	1034	
	miR-29c-5p	18	
miR-30	miR-30a-3p	1390	BECN1, BNIP3L, ATG12, ATG5, ATG2 (80); NFKB1 (82); E2F3, CCNE2, SKP2, CDK6, TFDP1, LDHA, GOT2, DNMT3B, ST6GALNAC1 (83); SOX2 - OT, PDK1 (84)
	miR-30a-5p	1539	
	miR-30b-3p	1207	
	miR-30b-5p	1545	
	miR-30c-1-3p	737	
	miR-30c-2-3p	743	
	miR-30c-5p	1545	
	miR-30d-3p	1390	
	miR-30d-5p	1539	
	miR-30e-3p	1396	
	miR-30e-5p	1541	
miR-126	miR-126-3p	19	ITGA6 (88)
	miR-126-5p	1362	
miR-134	miR-134-3p	501	FOXM1 (90); WWOX, CCND1, CCND2, CDK4, CASP3, Bcl-2, MMP9 (92); EGFR (93); MRP1 (94); DAB2 (95)
	miR-134-5p	452	
miR-144	miR-144-3p	1254	TIGAR (97); GLUT1 (98); ATF2 (99)
	miR-144-5p	240	
miR-145	miR-145-3p	361	CDK4 (101); SOX2, OCT4 (102); MTDH, EPN3, TPD52, CYP27B1, LMAN1, STAT1, TXNDC12 (104); TGFβ1 (105); RIOK2, NOB1 (106); VEGF (107); GOLM1, RTKN (108)
	miR-145-5p	909	
miR-150	miR-150-3p	413	FOXO4, E-cadherin, N-cadherin (110); GSKIP, β-catenin, HMGA2 (111); hLKB1 (112)
	miR-150-5p	902	
miR-182	miR-182-3p	388	FOXO3, N-cadherin, ADAM9, CDH9 (114); CASP2 (117); EPAS1 (118); IL-8, STAT3 (119)
	miR-182-5p	1266	
miR-185	miR-185-3p	843	IL-8, STAT3 (119); ABCC1 (121); EGFR-TKI, PFKL (122); YWHAZ (123)
	miR-185-5p	1137	
miR-198	​	448	FUT8 (127)

### 3.1 MiR-1-3p

The miR-1-3p is located within the intron region of the gene encoding the protein MIB E3 ubiquitin protein ligase 1 (MIB1), which is found on chromosome 18q11.2 (39). The observed downregulation of miR-1-3p expression in LUAD tissues and cell lines suggests a potential role in tumorigenesis. Conversely, the overexpression of miR-1-3p in LUAD cells led to a significant reduction in viability, migration, and invasion. The interaction of miR-1-3p with the 3′ untranslated region of the protein regulator of cytokinesis 1 (PRC1) resulted in their repression. In addition, elevated PRC1 expression has been shown to reverse the suppressive effects of miR-1-3p. This finding indicates that the miR-1-3p/PRC1 axis plays a pivotal role in the suppression of LUAD development and progression (40). Furthermore, Jiao et al. reported that the expression of the MET proto-oncogene, receptor tyrosine kinase (c-Met), could mitigate the effects of both miR-1-3p and miR-206 on hepatocyte growth factor (HGF) receptor-induced gefitinib resistance in LC (41). Finally, Roman-Canal et al. determined the expression of miR-1-3p in isolated extracellular vesicles (EVs) from 25 normal PE and 21 pleural lavages from patients with LC. The findings indicated an average accuracy of 94.1%, sensitivity of 92.9%, specificity of 95.0%, and an AUC value of 0.914. Concurrently, the expression of miR-1-3p was downregulated 13-fold in patients with LC compared with the control group (31).

### 3.2 MiR-19a/MiR-19b

The miR-19 family encompasses three members: miR-19a, −19b-1, and -19b-2. These miRNAs are derived from two distinct clusters. The miR-17-92 cluster has been demonstrated to regulate the expression of miR-19a and miR-19b-1. The miR-106a-363 cluster, located on chromosome X, encodes miR-19b-2 (42). In LUAD cells, the overexpression of miR-19a and miR-19b-1 has been shown to increase the epithelial-mesenchymal transition (EMT). Conversely, studies have demonstrated that the suppression of miR-19 can reverse EMT, thereby reducing migration and invasion (43, 44). Additionally, elevated levels of miRNA-19a in the peripheral blood of patients with NSCLC have been demonstrated to be associated with a poor prognosis. This finding suggests a potential correlation between this phenomenon and MAX dimerization protein 1 (MXD1), a protein involved in proliferation and metastasis (45). Furthermore, the microtubule-associated tumor suppressor 1 (MTUS1) gene has been implicated in the proliferation and migration of LUAD cells (46).

The interferon (IFN)-mediated innate immune response constitutes an integral component of the body’s primary defense system against pathogens. Li et al. established that the expression of miR-19a and miR-19b-1 was associated with a significant decrease in the expression of IFN-regulated genes, including interferon regulatory factor 7 (IRF7), interferon alpha inducible protein (IFI6), interferon-induced transmembrane protein 1 (IFITM1), interferon alpha inducible protein 27 (IFI27), and interferon-induced protein 44 like (IFI44L) in LC cells. Furthermore, the expression of miR-19a or miR-19b-1 has been observed to result in a decrease in the expression profile of major histocompatibility complex (MHC) class I genes, including MHC class I (B, E, F, G) (42). Furthermore, an evaluation of 29 MPELUM and 24 RMC samples revealed that miR-19a, miR-19b, and miR-21 exhibited significant upregulation, while miR-126 demonstrated notable downregulation. In accordance with the guidelines established by the International Mesothelioma Interest Group, the implementation of a combination of both miR-19a and miR-19b as diagnostic markers for LUM exhibits a sensitivity or specificity greater than 80% (24).

### 3.3 MiR-21

The gene encoding miRNA-21 is located on the 17th chromosome (17q.23.1) and is found within the 11th intron of the transmembrane protein 49 (TMEM49) gene. The transcription of the miR-21 gene results in the formation of a pre-miR-21 molecule that is 3,433 nucleotides in length. Subsequently, the 72-nt-long pre-miRNA loop is cleaved, resulting in the formation of two mature miRNAs: miR-21-5p and miR-21-3p (47). Seike et al. describe a significant correlation between phosphorylated-epidermal growth factor receptor (p-EGFR) levels and miR-21 expression in LC cells, suggesting that the EGFR signaling pathway positively regulates miR-21 expression. In addition, the antisense inhibition of miR-21 was demonstrated to promote apoptosis (48). As indicated in the study by Wei et al. the expression of miR-21 was found to be increased in the plasma of patients with NSCLC when compared to healthy subjects. In a cohort of 35 patients who received chemotherapy comprising cisplatin or carboplatin, 11 patients exhibited a partial response (PR). Plasma miR-21 expression levels were found to be significantly lower in patients who achieved a PR compared to those who did not respond and were comparable to levels observed in healthy controls (49). As demonstrated by Ni et al. the upregulation of miR-21 promoted cell migration and cell growth in NSCLC. This upregulation was associated with enhanced intracellular contents of lipids, including phospholipids, neutral lipids, and triglycerides. The levels of key lipid metabolic enzymes, including fatty acid synthase (FASN), acetyl-CoA carboxylase 1 (ACC1), and fatty acid-binding protein 5 (FABP5), as well as miR-21, were directly targeted by peroxisome proliferator-activated receptor gamma coactivator 1 beta (PPARGC1B) (50).

In addition, Xu et al. discovered an elevated expression of miR-155 and miR-21 in the serum of NSCLC patients when compared with the control group. This elevated expression was also associated with recurrence and metastasis (51). In a similar finding, Lara et al. revealed that the absence of miR-21 in LUAD-A549 cells (KO-A549) led to a decrease in proliferation, migration, and colony formation. Furthermore, the expression levels of phosphatase and tensin homolog (PTEN) and programmed cell death 4 (PDCD4) were found to be elevated in the KO-A549 cells in comparison to the A549 wild type. In addition, a decline in the inhibitory concentration 50% (IC_50_) values of drugs, including gemcitabine, carboplatin, paclitaxel, and oxaliplatin, has been observed (52). Finally, Liu et al. recognized that DNA ploidy analysis (DPA) exhibited a higher degree of accuracy in MPE diagnosis compared to exfoliative cytology in 40 patients diagnosed with LSC and NSCLC. Consequently, the enhanced expression of miR-21 and miR-24 was observed in MPE compared to BPE. The combination of DPA and miR-21 has been identified as a biomarker that differentiates MPE from BPE (27).

### 3.4 MiR-22

MiR-22 is located on chromosome 17p13. Its cDNA, catalyzed by RNA polymerase II, is approximately 1.3 kb. It has been determined that the promoter transcription start site is devoid of a TATA box (53). Zhang et al. found a decrease in miR-22 expression and a notable increase in levels of Snail family transcriptional repressor 1 (SNAI1) in LUAD cells compared to non-malignant cells. Furthermore, LUAD cells demonstrated a significant enhancement in invasive properties and the capacity for colony formation. The transforming growth factor-beta 1 (TGFβ1) has been observed to reduce miR-22 and E-cadherin while increasing Snail and N-cadherin (54). In addition, Gu et al. discovered that miR-22 is highly expressed in endothelial cells (ECs) and is downregulated in NSCLC tissues when compared to non-malignant tissues. This decline in miR-22 expression is potentially induced by interleukin-1b (IL1B), which is secreted by NSCLC cells and subsequently activates transcription factor nuclear factor-kB (NFKB1). Endothelial miR-22 has been identified as a significant inhibitor of angiogenesis, a process that plays a crucial role in cell growth. Direct targeting of sirtuin 1 (SIRT1) and fibroblast growth factor receptor 1 (FGFR1) in ECs has been demonstrated to inactivate the AKT/mammalian target of rapamycin (mTOR) signaling pathway (55).

The study by Yang et al. found a decrease in miR-22-3p expression in LC tissues compared to normal lung tissues. Additionally, miR-22-3p mimics, as well as the decline in c-MET and signal transducer and activator of transcription 3 (STAT3) protein expression, repress the capacity for colony formation and cell proliferation of LC cells. Concurrently, this treatment induced apoptosis, as indicated by the activation of poly (ADP-ribose) polymerase 1 (PARP) protein (56). According to Shin et al. the cohort of 87 patients was diagnosed with PE (45 cases of MPELUAD and 42 cases of BPE). The expression level of miR-22 in MPELUAD samples was found to be lower compared to that observed in the BPE samples. The diagnostic performance of the three combined miRNAs (−134, −185, and −22) with an AUC of 0.893 was comparable to that of the Carcinoembryonic Antigen (CEA) with an AUC of 0.898 (22).

### 3.5 MiR-24

The miR-23 ∼27 ∼24 family is comprised of multiple members and two paralogs: miR-23a ∼27a ∼24-2 (miR-23a cluster) and miR-23b ∼27b ∼24-1 (miR-23b cluster) on chromosome 9. A comparison of the mature sequences of miR-23a and miR-27a reveals a single nucleotide difference compared to their corresponding paralogs, miR-23b and miR-27b (57). Furthermore, Franchina et al. discovered that NSCLC patients who presented progressive disease exhibited a substantial upregulation of miR-22 expression. The correlation levels of miR-22 in whole blood and the absence of response in patients with pemetrexed-treated NSCLC suggest that the high levels of circulating miR-22 are a novel predictive biomarker for pemetrexed-based treatment (58). Finally, Wang et al. reported that the inhibition of miR-24 induces apoptosis by activating caspase 3 (CASP 3), thereby suppressing the viability and proliferation of NSCLC cells. The downregulation of miR-24 has been demonstrated to impede the invasive potential of NSCLC cells by modulating metalloproteinase 9 (MMP9). Furthermore, WW domain-containing oxidoreductase (WWOX) has been identified as a functional target of miR-24. Consequently, miR-24 may function as an oncogene, contributing to cell growth and migration (59).

Liu et al. reported that the expression of miR-24 exhibited a marked increase in NSCLC tissues compared to non-malignant tissues. The overexpression of miR-24 has been demonstrated to promote cell migration and invasion. In addition, zinc finger protein 367 (ZNF367) was identified as a downstream target of miR-24 (60). As indicated by Liu et al. the total number of subjects included in the study was 40 patients (20 cases of MPE/20 cases of BPLE). In the MPE group, DPA exhibited a higher rate of accuracy in MPE diagnosis. Furthermore, the miR-24 expression was elevated in MPE compared to BPE. The AUC for the combination of miR-24 and DPA was 0.973, with sensitivity and specificity of 100% and 80%, respectively. Consequently, the integration of DPA/miR-24 serves as a biomarker that differentiates MPE from BPE (27). Finally, Xie et al. reported that 110 subjects (28 BPE/82 MPE) exhibited significantly higher levels of miR-24 expression in the MPE compared to the BPE group. The AUC for miR-24 was 0.71 (95% confidence interval [95% CI] 0.578–0.820) (37).

### 3.6 MiR-25

The cluster (miR-106b, miR-93, and miR-25) is located in a 515 base pair region on chromosome 7q22 in intron 13. The mature miRNA molecule, designated as miR-25-3p, consists of a 22-nucleotide sequence and is classified as part of the evolutionary broadly conserved miR25-3p/32-5p/92-3p/363-3p/367-3p seed family (61). Xu et al. reported that plasma samples from 100 female non-smoking patients with LUAD contained significantly elevated levels of miR-25. The analysis of tissue samples revealed a significant association between elevated levels of plasma miR-25 and lymph node metastasis, advanced clinical stage, and EGFR mutation (62). Furthermore, Wu et al. observed the miR-25 expression in 51 cases of LUAD, 30 cases of LSC, and 41 healthy subjects. The plasma of patients diagnosed with LC demonstrated elevated levels of miR-25, which plays a crucial role in regulating gene expression. However, the inhibition of miR-25 resulted in a substantial decrease in cell proliferation and apoptosis. The modulator of apoptosis 1 (MOAP1) gene has been identified as a novel target of miR-25 (63). As indicated by Ding et al. the expression of miR-25 was found to be significantly elevated in 31 samples of NSCLC in comparison to that observed in non-malignant tissues. This elevated expression was identified as a novel gene of miR-25, the Factor 4 Kruppel-like (KLF4). Furthermore, the suppression of KLF4 has been observed to promote cell migration and invasion, while the restoration of KLF4 expression led to a reduction in cell motility in miR-25 in NSCLC cells. The activation of the extracellular signal-regulated kinase (ERK) signaling pathway by miR-25 ultimately resulted in elevated levels of vimentin, MMP11, and N-cadherin, concurrent with the repression of E-cadherin expression through the inhibition of KLF4 expression (64).

In a cohort of 113 patients with NSCLC, the upregulation of miR-25 has been demonstrated to be associated with lymphatic metastasis. Furthermore, the overexpression of miR-25 enhanced migration and invasion, but not apoptosis and proliferation, by inactivating cadherin 1 (CDH1) expression. Consequently, patients exhibiting elevated levels of miR-25 are predisposed to lymph node metastasis and a poor prognosis (65). In addition, Zhang et al. detected an upregulation of plasma miR-25 in 114 patients with NSCLC who exhibited positive lymph node metastasis, poor differentiation, or advanced clinical stage. In contrast, surgical intervention resulted in a decline in plasma miR-25 expression in 45 patients with NSCLC. The receiver operating characteristic (ROC) curve analysis indicated that the plasma miR-25 exhibited high diagnostic sensitivity and specificity in discriminating between NSCLC and healthy subjects (66).

In addition, Wu et al. reported that NSCLC patients exhibited elevated levels of miR-25, which was associated with diminished OS. However, these same patients demonstrated a propensity for cell migration and invasion when compared with those who exhibited reduced levels. The large tumor suppressor 2 (LATS2) gene was identified as a novel target of miR-25. The impact of miR-25 on the migration and invasion of NSCLC cells was attributable to the stimulation of the LATS2/YAP/Hippo signaling pathway (67). In a similar observation, Huang et al. detected that the miR-25 expression was significantly higher in patients with NSCLC who had peripheral infiltration, pathological grades, and tumor-node-metastasis (TNM) staging classifications, as well as with lymph node metastasis, when compared to patients without peripheral infiltration. Furthermore, patients demonstrating elevated levels of miR-25 exhibited a substantially diminished survival probability when compared with patients exhibiting reduced levels of this miRNA (68). As demonstrated by Lv et al. an elevated serum level of miR-25 was observed in 86 patients diagnosed with NSCLC in comparison to a control group. These increases were associated with advanced age (≥60 years), radiotherapy, histological type, low survival rate, and low median survival time. Furthermore, the upregulation of miRNAs (−130a, −25, and −191) has been demonstrated to promote invasiveness and metastasis (69).

### 3.7 MiR-29c

The precursors of the miR-29 family, particularly miR-29b-1 and miR-29a, have been identified as residing on chromosome 7q32.3. Concurrently, the precursors of miR-29b-2 and miR-29c are situated on chromosome 1q32.2. Recent findings indicate that miR-29b-2 and miR-29c are encoded by the final exon of the primary miR-29b-2/c transcript. In contrast, the precursors of miR-29b-1 and miR-29a are processed from the last intron of the primary transcript EU154353 (70). According to Wang et al. an elevated expression of miR-29c in high-metastatic LC 95D cells led to a decrease in cell proliferation, adhesion to the extracellular matrix, invasion, and migration. Conversely, the loss-of-function that reduced miR-29c using its inhibitor in 95C cells promoted proliferation, adhesion to ECM, invasion, and migration. In addition, the expression of integrin b1 (ITGB1) and MMP2 was suppressed by miR-29c (71).

The research by Zhu et al. revealed that serum levels of miR-29c exhibited an increase, while levels of serum miR-429 demonstrated a decrease in patients with NSCLC. In addition, an association was observed between the levels of miR-29c and miR-93 and diminished OS (72). Likewise, Zhang et al. found that the miR-29c expression decreased in LC tissues while the expression of specificity protein 1 (Sp1) increased. Additionally, the expression of miR-29c was found to be downregulated in high-metastatic LC cell lines and in cells treated with transforming growth factor-beta 1 (TGF-β1). The study demonstrated that the inhibition of miR-29c or the induction of Sp1 enhanced cell migration and invasion. However, the expression of thyroid transcription factor 1 (TTF-1) and E-cadherin decreased, while vimentin and α-smooth muscle actin (α-SMA) increased (73). Furthermore, Liu et al. discovered that the downregulation of miR-29c expression was markedly associated with an unfavorable prognosis in patients with LUAD. *In vitro*, miR-29c has been shown to exert a suppressive effect on various hallmarks of cancerous cell behavior, including cell proliferation, migration, and invasion. Integrative analysis recognized vascular endothelial growth factor (VEGF) as a direct target of miR-29c (74).

As indicated by Arechaga-Ocampo et al. the Calu-1 cells of NSCLC exhibited a high degree of resistance and suppression of miR-29c. The restoration of miR-29c expression has been demonstrated to reverse the radioresistance by inducing apoptosis and downregulating Bcl-2 and Mcl-1 genes (75). Furthermore, Sun et al. discovered that the miR-29c expression in tissues from patients with NSCLC. In contrast, the administration of cisplatin increased in the miR-29c expression and a concomitant decrease in the expression of its oncogenic target, protein kinase (Akt). This finding suggests that elevated levels of miR-29c are associated with prolonged disease-free survival, indicating a potential role in the progression and management of the disease. Furthermore, patients with recurrence following cisplatin chemotherapy exhibited a lower expression of miR-29c, suggesting that it may play a role in the resistance of NSCLC cells to cisplatin. Its mechanism of action involves the negative regulation of the phosphoinositide-3-kinase (PI3K) pathway (76).

As reported by Zhan et al. the expression of miR-29c was found to be lower in NSCLC tissues compared to normal tissues. A negative correlation was identified between the expression levels of miR-29c and VEGF levels. Conversely, ectopic expression of miR-29c has been demonstrated to impede cell proliferation and apoptosis (77). In the LC cachexia model, it was demonstrated that the expression of miR-29c was reduced, and its expression was inversely correlated with muscle catabolic activity. Mechanistic studies revealed that leukemia inhibitory factor (LIF) was a direct target of miR-29c (78). Finally, in serum and MPE, the levels of miR-29c decline in LC patients compared to BPE patients. However, comparative analysis revealed that the serum and PE levels of miR-29c in BPE patients were higher compared with MPE patients (34).

### 3.8 MiR-30d

MiR-30d-5p is located on chromosome 8q24.22. The identified miRNA cluster was found to be composed of miR-30b/d, with the seed family of miR-30d-5p comprising miR-30abcdef/384–5p. MiR-30d-5p is subject to regulation by LINCRNA (79). It has been proposed that miR-30d may modulate the expression of multiple genes within the autophagy pathway, including beclin 1 (BECN1), Bcl-2 interacting protein 3 like (BNIP3L), autophagy-related 12, 5, and 2 (ATG12, ATG5, and ATG2) (80). Hosseini et al. found that the miR-30d expression exhibited a substantial decrease in resected tissue samples from patients with NSCLC compared to the control group. The observations suggest the possibility of a tumor-suppressing function for miR-30d in the progression and initiation of LC (81). As demonstrated in the study by Wu et al. the overexpression of miR-30d was found to result in a significant inhibition of cell migration and invasion in NSCLC cell lines. Mechanistically, miR-30d functions as a negative regulator of nuclear factor I B (NFIB) by directly targeting its 30-untranslated region. Conversely, NFIB reverses the suppressive effects of miR-30d on cell migration and invasion (82).

As indicated by Chen et al. in patients with NSCLC exhibiting lower levels of miR-30d-5p, there was an advanced clinical progression and the presence of implication in the process, including the mucin-type O-glycan biosynthesis pathway, the cell cycle pathway, and the cysteine and methionine metabolism pathway. Consequently, it can be hypothesized that miR-30d-5p may possess the capacity to impede the proliferation and viability of NSCLC cells. A total of nine genes were identified as probable targets of miR-30d-5p: E2F transcription factor 3 (E2F3), cyclin E2 (CCNE2), S-phase kinase-associated protein 2 (SKP2), cyclin-dependent kinase 6 (CDK6), transcription factor Dp-1 (TFDP1), lactate dehydrogenase A (LDHA), glutamic-oxaloacetic transaminase 2 (GOT2), DNA methyltransferase 3 beta (DNMT3B), and ST6 N-acetylgalactosaminide alpha-2,6-sialyltransferase 1 (ST6GALNAC1) were recognized as probable targets of miR-30d-5p (83).

As demonstrated by Chen et al. the SOX2 overlapping transcript (SOX2-OT) and pyruvate dehydrogenase kinase 1 (PDK1) exhibited significant upregulation, while miR-30d-5p demonstrated notable downregulation in NSCLC. The overexpression of PDK1 or the inhibition of miR-30d-5p expression has been shown to reverse the inhibitory effect of SOX2-OT silencing on malignant progression (84). In addition, Xie et al. identified elevated levels of miR-30d in 110 subjects with LC (82 cases of MPE/28 cases of BPE), demonstrating higher levels in the malignant group compared to the control group (37).

### 3.9 MiR-126

MiR-126 is encoded by intron 7 of the epidermal growth factor-like domain-containing gene 7 (EGFL7) on human chromosome 9q34.3. MiR-126 has been demonstrated to function as a silencer of genes such as mTOR and phosphoinositide-3-kinase regulatory subunit 2 (PIK3R2), thereby modulating the expression of the EGFL7 isoform B (85). As the research by Yang et al. has demonstrated, the overexpression of miR-12 in NSCLC cells has been shown to reduce cell proliferation. The mechanism of action of miR-126 involves the repression of the PI3K-Akt pathway by targeting binding sites in the 39 untranslated regions of PI3KR2 mRNA. Patients exhibiting low levels of miR-12 demonstrated a reduced survival rate (86).

Conversely, Grimolizzi et al. observed a decrease in the serum miR-126 expression in 45 patients with NSCLC. In contrast, healthy controls exhibited an equal distribution of circulating miR-126 between exosome- and exosome-free serum fractions. Additionally, the incubation of exosome samples derived from patients with early- and advanced-stage NSCLC resulted in the formation of blood vessels and malignant transformation. Furthermore, the integration of human bronchial epithelial exosome-enriched miR-126 from normal endothelial cells into NSCLC cells resulted in the suppression of cell growth and the promotion of malignancy (87). Concurrently, Li et al. discovered that in 20 patients with NSCLC, serum-derived exosome analysis exhibited low levels of miR-126 expression, which was associated with increased cell proliferation, cell cycle progression, cell migration, and invasion. In addition, the incorporation of a mimic of miR-126 into vesicle-derived serum from NSCLC has been demonstrated to attenuate cell proliferation, colony formation, migration, and invasion, while concomitantly inducing cell cycle arrest and apoptosis. The underlying mechanism involves integrin subunit alpha 6 (ITGA6) as a target of miR-126 (88).

### 3.10 MiR-134

MiR-134 is located on chromosome 14q32. The presence of differentially methylated regions has been detected within the DLK1-DIO3 region. MiR-134 has been identified as a regulator of dendritic spine development. Its mechanism of action involves the targeting of Limk1 mRNA in rat hippocampal neurons (89). As Li et al. have demonstrated, the expression of miR-134 impedes the process of EMT in NSCLC cells. Additionally, the Forkhead box M1 (FOXM1) protein has been identified as a direct target of miR-134. The suppression of FOXM1 resulted in the reversion of EMT, which is analogous to the effects observed in cells with overexpressed miR-134 (90).

As indicated by Chen et al. the expression of miR-134 has been associated with the proliferation of LSC cells and the suppression of their apoptosis. Furthermore, the transfection of miR-134 mimics into LSC cells led to a significant decrease in WWOX levels. This decline in WWOX expression was found to be a crucial factor in the promotion of cell proliferation, and the suppression of apoptosis by miR-134 occurred via activation of the extracellular signal-regulated kinase 1/2 (ERK1/2) pathway (91). Sun et al. reported a substantial decline in the expression of miR-134 in tissues derived from NSCLC patients. Furthermore, ectopic expression of miR-134 led to a significant suppression in cell growth, achieved through the inhibition of cyclin D1 and D2 (CCND1, CCND2), cyclin-dependent kinase 4 (CDK4), and the upregulation of p57(Kip2) and p21(Waf1/Cip1). The induction of apoptosis by miR-134 was concomitant with the upregulation of cleaved caspase-3 (CASP3) and the downregulation of Bcl2. Furthermore, studies have revealed that miR-134 can impede cellular migration and invasiveness by selectively targeting the MMPs −7 and −9. Additionally, the oncogene CCND has been identified as a potential target of miR-134 (92).

Similarly, Qin et al. demonstrated that the expression of mi-134 impedes epidermal growth factor receptor (EGFR)-related signaling and suppresses the proliferation of NSCLC cells by inducing cell cycle arrest and apoptosis. Consequently, miR-134 has been identified as a tumor suppressor (93). As Li et al. have noted, the expression of miR-134 is reduced in A549/cisplatin multidrug resistance LUAD cells in comparison with A549 parental cells. MiR-134 has been shown to modulate the sensitivity of LUAD cells to specific anticancer drugs. Additionally, FOXM1 and multidrug resistance-associated protein 1 (MRP1) have been identified as direct targets of miR-134 (94). The research conducted by Zhang et al. detected elevated levels of miR-134-5p, concomitant with a decline in DAB adaptor protein 2 (DAB2) levels. These findings exhibited a substantial correlation with the recurrence of stage I LUAD patients. In addition, the overexpression of miR-134-5p or the silencing of DAB2 genes significantly promoted metastasis and chemoresistance (95).

### 3.11 MiR-144-5p

The miR-144 gene is located on chromosome 17q11.2. It is located 100 bp upstream of the miR-451 gene. It is also 40 bp downstream of the miR-4732 gene. Human RNase III (Dicer) facilitates the process of cytoplasmic biogenesis of pre-mir-144 into mature mir-144 (96). The TP53-induced glycolysis and apoptosis regulator (TIGAR) plays a pivotal role in the regulation of cellular processes, including proliferation, apoptosis, and autophagy. *In vitro*, studies have demonstrated that miR-144 exerts its tumor-suppressing function by targeting TIGAR, thereby impeding cell proliferation and inducing apoptosis and autophagy (97).

Conversely, glucose uptake rate and lactate production assays demonstrated that the miR-144 expression decreased, thereby enhancing the aerobic metabolism of LC cells. The miR-144 expression increases the expression level of glucose transporter 1 (GLUT1), which leads to an increase in glucose uptake and lactate production. This process has been linked to rapid cell proliferation (98). Another study found that NSCLC tissues and cell lines exposed to radiation exhibited diminished levels of miR-144-5p expression. As has been demonstrated, the transcription factor 2 (ATF2) is directly targeted by miR-144-5p. It is also a functional target (99). In a recent study, Roman-Canal et al. presented findings that indicated the presence of EVs in 25 patients diagnosed with BPE and 21 patients with MPE who had LC. The analysis revealed that miR-144-5p exhibited an AUC of 0.882 and a sensitivity of 78.6% (31).

### 3.12 MiR-145

MiR-145 is located on chromosome 5q32-33 and is 4.08 kb in length. The miR-145 locus can generate two transcripts: miR-145-3p and miR-145-5p. The processing of the microRNA (miRNA) transcript yields approximately 22 nucleotides, while the miR-145-5p transcript generates 23 nucleotides (100). As indicated by Chen et al. a decline in the expression of miRNA-145 has been detected in NSCLC tissues and cell lines. The miR-145 has been shown to impede the c-Myc/eIF4E pathway, which plays a crucial role in regulating cellular proliferation. Furthermore, CDK4 is subject to regulation by miR-145 within the cell cycle control (101). Conversely, the expression of miR-145 has been observed to regulate the translation of SRY-related HMG box (SOX2) and octamer-binding transcription factor (OCT4), while the p53 protein has been shown to regulate the expression of miR-145. The low levels of miRNA-145, in conjunction with p53 mutations, have been identified as independent markers of a reduced time to relapse in patients with LC (102).

Concurrently, Li et al. discovered that the expression of miR-145 was diminished in LUAD cases accompanied by positive lymph node metastasis, while the expression of miR-10b exhibited an increase. The expression levels of these miRNAs have been demonstrated to be associated with the presence of lymph node metastasis in cases of NSCLC. However, the suppressive effect of miR-145 on migration and invasion was observed (103). As Mataki et al. have demonstrated, a decline in the levels of miRNAs has been observed. The presence of -145-5p and -145-3p was identified in the LC tissues. In addition, seven putative target genes, including metadherin (MTDH), epsin 3 (EPN3), tumor protein D52 (TPD52), cytochrome P450 family 27 subfamily B member 1 (CYP27B1), lectin, mannose-binding 1 (LMAN1), signal transducer and activator of transcription 1 (STAT1), and thioredoxin domain containing 12 (TXNDC12), were found to be regulated by miR-145-5p and miR-145-3p in LSC. The elevated MTDH expression was a reliable predictor of diminished survival outcomes in patients diagnosed with LSC (104). In a similar vein, Yin et al. observed that the levels of miR-145 and miR-497 were diminished in NSCLC cells. The ectopic expression of these miRNAs significantly impeded the TGF-β1-induced EMT and reduced the migration and invasion. The miR-145 and miR-497 have been shown to attenuate MTDH expression through direct binding to the 3′-untranslated region of MTDH mRNA (105).

As Liu et al. have observed, right open reading frame kinase 2 (RIOK2) and NIN1 (RPN12) binding protein 1 homolog (NOB-1) have been identified as critical accessory factors in ribosome assembly. In the context of NSCLC, research findings have indicated that the expression of ROK2 and NOB1 is subject to inhibition in the presence of overexpression of miR-145. As indicated by the aforementioned findings, the promotion of the suppression of cell viability, migration, and invasion is a key factor I in the observed outcomes (106). In contrast, low serum levels of miR-145 were associated with poor OS, while high levels of VEGF did not demonstrate a similar association. *In vitro*, the expression of miR-145 has been shown to induce a state of cell cycle arrest. The combination of miR-145 and VEGF levels has the potential to serve as a diagnostic tool for NSCLC (107).

A comparative analysis reveals that the introduction of miR-145 into NSCLC cells impedes their proliferation, migration, and invasion. In addition, evidence indicates that Golgi membrane protein 1 (GOLM1) and Rhotekin (RTKN) are direct targets of miR-145, suggesting a regulatory role in disease progression (108). Huang et al. reported that the cohort of LC patients included 49 cases of MPE and 51 cases of BPE. The sensitivity and specificity of miR-145 were determined to be 64.71% and 79.59%, respectively. In contrast to this finding, the sensitivity and specificity of the CD276 molecule (B7-H3) were found to be 80.39% and 61.22%, respectively. It is crucial to recognize the significance of B7-H3 and miR-145 in relation to lymphatic metastasis, differentiation degree, and TNM stage (38).

### 3.13 MiR-150-5p

MiR-150-5p, localized to chromosome 19q13, has been detected as a potential biomarker for metastatic cancers. The potential of miR-150 to modulate the EMT is an important phase in the process of tumor cell migration and metastasis (109). According to Li et al. the critical pro-metastatic role of miR-150 in the regulation of EMT is facilitated by repression of forkhead box O4 (FOXO4) in NSCLC. Furthermore, ectopic expression of miR-150 enhanced tumor cell metastasis and triggered EMT-like changes in NSCLC cells (including E-cadherin repression, N-cadherin and vimentin induction, and mesenchymal morphology). Conversely, the knockdown of FOXO4 exhibited pro-metastatic and molecular effects analogous to those observed in the overexpression of miR-150 (110).

In addition, Dai et al. observed that the inhibition of miR-150-5p has exerted several effects on cancer stem cells (CSC). The inhibition of miR-150-5p has been demonstrated to result in an increase in the population of CSC and the formation of spheres. Moreover, ectopic expression of miR-150-5p has been demonstrated to impede the formation of spheres in NSCLC and metastasis. Furthermore, the miR-150-5p was found to inhibit Wingless (Wnt) by simultaneously targeting glycogen synthase kinase 3 beta interacting protein (GSKIP) and β-catenin. This regulatory mechanism involves the targeting of high mobility group AT-hook 2 (HMGA2), a critical component in these regulatory pathways (111). In a related study, Wu et al. discovered elevated levels of miR-150-5p expression in NSCLC tissues and cell lines. The miR-150-5p promotes cellular proliferation and migration while concomitantly reducing cellular apoptosis. In contrast, the inhibition of miR-150-5p resulted in the suppression of cellular growth, which was found to be regulated by serine/threonine kinase 11 (hLKB1) (112). As Roman-Canal et al. have noted, EVs were isolated from 25 PE and 21 pleural lavages from patients with LC. The AUC of miR-150-5p was 0.912, and its sensitivity was 85.7%, with a 3-fold expression in patients with LC. The EV-associated miRNAs from PE and lavages have led to the identification of biomarkers, including miR-1-3p, miR-144-5p, and miR-150-5p (31).

### 3.14 MiR-182

The chromosomal order of miRNAs is well conserved in deuterostomes (miR-183, miR-96, and miR-182). In humans, the cluster is localized on chromosome 7, accompanied by an intergenic region measuring 4.2 kb between miR-96 and miR-182 (113). According to Yang et al. the expression of Sp1 increases in the transcriptional activity of forkhead box O3 (FOXO3). Sp1 has been shown to enhance the expression of miR-182, and this increased expression has been demonstrated to be recruited to the 3′untranslated region (3′UTR) of FOXO3 mRNA. The suppression of miR-182 led to the inhibition of LC cell proliferation while concurrently enhancing the invasion and migration of these cells through the expression of N-cadherin. The suppression of FOXO3 expression in the miR-182 knockdown cells led to a partial reversal of this effect. This finding indicates that miR-182 may play a role in promoting cancer cell growth and metastatic activity. The expression levels of multiple genes associated with cancer metastasis, including ADAM9, CDH9, and CD44, exhibited an increase following the implementation of a miR-182 knockdown (114).

Similarly, Chen et al. found that the expression of miR-182 was overexpressed in LC. Moreover, the knockdown of miR-182 resulted in the suppression of cell proliferation and apoptosis subsequent to irradiation (115). Likewise, Zhu et al. observed elevated serum levels of miR-182, miR-183, and miR-210, while the level of miR-126 demonstrated a significant decrease in NSCLC patients compared with healthy controls. The relative levels of these serum miRNAs exhibited a high degree of sensitivity and specificity in distinguishing between early-stage NSCLC and current tobacco smokers without LC or pneumonia (116). Yang et al. found elevated levels of miR-182-5p expression in both the tissue and peripheral blood samples of NSCLC patients. The suppression of miR-182-5p has been demonstrated to promote the inhibition of cell proliferation and the induction of cell apoptosis. In contrast, a decline in CASP2 expression was observed in both NSCLC tissue and peripheral blood samples (117).

Furthermore, Yang et al. reported that the expression of miR-182-5p is elevated in tumor tissues and recognized as a prognostic factor for patients with NSCLC. The endothelial PAS domain-containing protein 1 (EPAS1) was identified as a direct target of miR-182-5p (118). As indicated in the report by Zhao et al. a significant upregulation of miR-182 was detected in bone-metastatic NSCLC cells and tumor samples. MiR-182 induces the secretion of interleukin-8 (IL-8) by NSCLC cells. This, in turn, has been observed to facilitate osteoclastogenesis via the activation of signal transducer and activator of STAT3 (119). Finally, Zhu et al. describe that the expression of miR-182 in serum and PE samples was elevated in MPE subjects than in BPE healthy patients. The expression levels of miR-21, miR-29c, and miR-182 have been shown to possess high diagnostic efficacy in differentiating patients with MPE from those with BPE (34).

### 3.15 MiR-185

The gene is located on chromosome 22q11.21. The precursor of the miRNA is composed of 82 nucleotides, from which the two mature molecules, miR-185-5p and miR-185-3p, are derived (120). Pei et al. reported that the expression of miR-185-5p in A549 cells was found to be elevated in comparison to the levels observed in the A549/DDP cell line. The transfection of A549 cells with miR-185-5p mimics resulted in an increased sensitivity to cisplatin. The current study identified the ATP-binding cassette subfamily C member 1 (ABCC1) as the target gene of miR-185-5p (121). In addition, Li et al. recognized that the expression of miR-185-3p was downregulated, contributing to the development of acquired resistance to epidermal growth factor receptor tyrosine kinase inhibitors (EGFR-TKI) in LC cells. Mechanistically, the downregulation of miR-185-3p contributed to the endoplasmic reticulum resistance by upregulation of phosphofructokinase, liver type (PFKL). In addition, the investigation revealed that the MET facilitates resistance to EGFR-TKI by modulating miR-185-3p and PFKL (122).

Concurrently, Ma et al. noted a decline in the expression of miR-185-5p in NSCLC cell lines. The overexpression of miR-185-5p led to a decline in cell viability, proliferation, invasion/migration, and induction of cell apoptosis while concurrently inhibiting tumor growth. Furthermore, the direct binding interaction between tyrosine 3-monooxygenase/tryptophan 5-monooxygenase activation protein zeta (YWHAZ) and miR-185-5p has been identified (123). As Liu et al. observed, the serum expression of miR-185 was lower in healthy controls than in patients with NSCLC. Furthermore, serum miR-185 exhibited superior diagnostic accuracy (124). Finally, Shin et al. found this phenomenon in a cohort of 87 patients (45 MPELUAD/42 BPE). A comparative analysis of the expression levels of miR-185 in samples from the MPELUAD revealed a lower expression level compared to that observed in samples from the BPE (22).

### 3.16 MiR-198-5p

The gene is located on chromosome 3q13.33. The expression level of miR-198-5p was found to be significantly higher in patients with early-stage tumors (I-II) compared to those with advanced-stage tumors (III-IV) in LSC (125). As Wang et al. found, the expression of miR-198-5p is lower in LUAD tissues compared to adjacent non-malignant tissues. A rising body of research has identified a correlation between the expression of miR-198-5p and various clinical and pathological characteristics, including age, the presence of blood vessel invasion, and metastasis (126). As Wang et al. observed, miR-198-5p was found to be under-expressed in NSCLC tissues and negatively correlated with tumor size, lymph node metastasis, and TNM stage. The expression of fucosyltransferase 8 (FUT8) was found to be elevated. Additionally, the repression of miR-198-5p expression directly led to the enhancement of cell migration, invasion, and EMT (127). Finally, Han et al. describe a significant decrease in the expression of miR-198 in MPELUAD. The cut-off values for miR-198, CEA, and CYFRA21-1 were determined to be 0.134 ng/mL, 7.6 ng/mL, and 41.9 ng/mL, respectively. The analysis of the data yielded an AUC value of 0.887 for miR-198, indicating a sensitivity of 71.1% and a specificity of 95.2% (21).

## 4 Clinical relevance of miRNA in the diagnostic of LC

MiRNAs have emerged as promising biomarkers for the diagnosis of pleural malignancies. A thorough review of the extant research efforts aimed at assessing the diagnostic potential of miRNAs in samples from patients with PE reveals the presence of several limitations. The absence of absolute specificity for miRNAs suggests that certain BPE results may be positive, potentially leading to unnecessary procedures, patient injury, and inadequate management. This emphasizes the importance of integrating miRNA testing with clinical expertise and other diagnostic approaches. In the diagnosis of malignancies with unclear etiology, particularly in cases where standard cytology yields inconsistent results, reliance on the identification of miRNAs is imperative (128).

In the present review, the evaluation of individual miRNAs in pleural fluid for malignancy yielded AUC values ranging from 0.59 to 0.93. However, it is noteworthy that values ≥ 0.90 are considered adequate for clinical use (Table 3). Several miRNAs must be combined with other biomarkers, such as CEA. In a particular study, the combination of CEA and carbohydrate antigen 15-3 (CA 15-3) in PF, utilizing cut-off points with 100% specificity, led to the identification of only 40% of cytology-negative MPE cases. The 41%, 40%, and 60% of patients with PE exhibited elevated pleural concentrations of CEA, CA 15-3, or one of the aforementioned markers, respectively (129). Accordingly, Watabe et al. identified a dataset from The Cancer Genome Atlas consisting of pleural lavage fluid from 41 cases. The study found that the levels of miRNA-21 in pleural lavage fluid were associated with positive cytology and pleural invasion in the primary sites. This finding indicates that the presence of miRNA-21 in pleural lavage fluid is a substantial diagnostic and prognostic factor. The study revealed that the presence of miRNA-21 can trigger a transformation of the mesothelium into a mesenchymal state. This process has the potential to establish premetastatic niches within the pleural cavity, thereby facilitating the dissemination of cancerous cells (130). A recent study by Zhao et al. utilized next-generation sequencing technology to analyze the pleural fluid exosomal miRNA profile in 5 MPE and 15 BPE cases. The combination of exosomal miR-182-5p and CEA has been demonstrated to slightly enhance the diagnostic accuracy of MPE, with an AUC of 0.91. This finding suggests that pleural miR-182-5p may have a role in the diagnosis of MPE. A more thorough examination of the functional role of miRNA-182-5p in pleural metastasis and tumor microenvironment interactions may provide insights with both diagnostic and therapeutic implications (131). In a subsequent study, Shin et al. measured the expression of three miRNAs in samples from 87 patients with pleural effusion, including 45 cases of LUAD-MPE and 42 cases of BPE. The expression levels of miRNA-22, miRNA-134, and miRNA-185 were found to be significantly lower in LUAD-MPE compared to BPE. The AUC values for miRNA-134, miRNA-185, miRNA-22, and CEA were 0.721, 0.882, 0.832, and 0.898, respectively. The combination of CEA with the three miRNAs enhanced the diagnostic performance, resulting in an AUC of 0.942, with a sensitivity of 91.9% and a specificity of 92.5% (22). The combination of CEA and miRNAs, a multi-marker strategy, has the potential to yield superior diagnostic accuracy in comparison with the utilization of individual biomarkers. Wang et al. conducted a study that revealed the presence of PE in 184 patients diagnosed with NSCLC and MPE. A total of 33 miRNAs were identified as being more than twofold altered by microarray in malignant effusions between the longer-survival and shorter-survival groups. Furthermore, levels of five specific miRNAs (−93, −100, −134, −151, and −345) were found to be significantly associated with overall survival. The findings of this study demonstrated that elevated levels of miRNA-100 and diminished levels of miRNA-93, miR-134, -151, and miR-345 were correlated with survival outcomes in both the training and validation cohorts (132). Additionally, Marqués et al. identified an enrichment of the discovered miRNAs in migration-related processes through bioinformatics analysis. It is noteworthy that the abundance of three miRNAs (−141-3p, −203a-3, and −200c-3p) effectively classified MPE with false-negative cytological examination results, suggesting the potential of these molecules to enhance diagnostic accuracy (133).

MiRNAs have been shown to play a crucial role in the development of cancer. Aberrant expression levels of these molecules have been observed in various types of human tumors, including LC. The available evidence indicates a correlation between altered expression of specific miRNAs and the gene expression regulation that is involved in the hallmarks of cancer (Table 5). The non-invasive biomarkers under consideration facilitate not only the diagnosis and prognosis of diseases but also serve as novel predictors of cancer treatment response and sensitivity. The selection of high-risk patients for adjuvant systemic therapies necessitates the identification of specific and reliable biomarkers, a process of paramount importance in the context of LC, given the fact that nearly 50% of NSCLC patients develop distant metastases following pulmonary resection. The advent of targeted therapies, predicated on the identification of specific mutations, has led to a marked enhancement in the management of LC (134). The specific expression patterns of miRNAs in patients with PE have been demonstrated to correlate with disease progression, response to treatment, and overall survival. Several miRNAs, including members of the let-7 family, as well as miR-34a and miR-21, are currently being investigated for their therapeutic potential. The implementation of RNA-based methodologies holds the promise of a paradigm shift in the management of LC, offering precise, personalized, and minimally invasive solutions for diagnosis and treatment (135).

**TABLE 5:** Overview of the genes and signaling pathways affected by miRNA expression in LC.

miRNA	Gene/signaling pathway: increased (+) or decreased (−) expression	Hallmarks cancer
miR-1-3p	PRC1 (−)	Viability, migration, and invasion
MiR-19a/MiR-19b	EMT (+), MMXD1 (+), IRF7 (−), IFI6 (−), IFITM1 (−), IFI27 (−), IFI44L (−), MHC (−)	Proliferation, migration, invasion, and metastasis
MiR-21	p-EGFR (+), PTEN (−), PDCD4 (−)	Proliferation, migration, apoptosis, metastasis, and drug resistance
MiR-22	SNAI1 (−), c-MET (−), STAT3 (−)	Proliferation, angiogenesis, invasion, and apoptosis
MiR-24	CASP 3 (+), MMP9 (+), WWOX (+), ZNF367 (−)	Viability, proliferation, apoptosis, drug resistance, migration, and invasion
MiR-25	MOAP1 (+), KLF4 (+), vimentin (+), MMP11 (+), N-cadherin (+), E-cadherin (−), LATS2 (+)	Migration, invasion, and metastasis
MiR-29c	ITGB1 (−), MMP2 (−), Sp1 (+),VEGF (+), Bcl-2 (−), Mcl-1 (−), Akt pathway (−), PI3K pathway (−), LIF (−)	Proliferation, adhesion, invasion, migration, and metastasis
MiR-30d	BECN1 (+), Bcl-2 (+), BNIP3L (+), ATG12 (+), ATG5 (+), ATG2 (+), NFIB (−), E2F3 (+), CCNE2 (+), SKP2 (+), CDK6 (+), TFDP1 (+), LDHA (+), GOT2 (+), DNMT3B (+), ST6GALNAC1 (+), SOX2-OT (+), PDK1 (+)	Autophagy, migration, and invasion
MiR-126	mTOR pathway (−), PIK3R2 (−), PI3K-Akt pathway (−), ITGA6 (+)	Proliferation, migration, invasion, cell cycle arrest, and apoptosis
MiR-134	EMT (−), FOXM1 (+),WWOX (−), CCND1 (−), CCND2 (−), CDK4 (−), Waf1/Cip1 (−), CASP3 (+), Bcl2 (−), MMP7 (−), MMP9 (−), EGFR) (−), MRP1 (+), DAB2 (−)	Drug resistance, proliferation, apoptosis, migration, and invasion
MiR-144-5p	TIGAR (+), GLUT1 (+), ATF2 (+)	Proliferation, apoptosis, and autophagy
MiR-145	c-Myc/eIF4E pathway (−),CDK4 (+),SOX2 (+), OCT4 (+), MTDH (+), EPN3 (+), TPD52 (+), CYP27B1 (+), LMAN1 (+), STAT1 (+), TXNDC12 (+), GOLM1 (+), RTKN (+)	Proliferation, metastasis, migration, and invasion
MiR-150-5p	EMT (+), FOXO4 (−), E-cadherin (−), N-cadherin (+), vimentin (+), Wnt (−), GSKIP (−), β-catenin (−), hLKB1 (−)	Metastasis, proliferation, migration, and apoptosis
MiR-182	FOXO3 (−), ADAM9 (−), CDH9 (−), CD44 (−), CASP 2 (−), EPAS1 (+)	Proliferation, invasion, migration, and metastasis
MiR-185	ABCC1 (+), EGFR-TKI (−), PFKL (+)	Drug resistance, proliferation, invasion, migration, and apoptosis
MiR-198-5p	FUT8 (+), EMT (+)	Migration and invasion

## 5 Conclusion and perspectives

The development of new molecular biomarkers that facilitate early diagnosis, as evidenced by the case of LC, is necessary to enhance patient survival. Several biomarkers are available as possible candidates; nevertheless, there are limitations regarding access to biological samples due to the invasiveness of the procedures. In recent research, considerable attention has been directed toward transcriptional factors/non-coding RNAs, including miRNAs. The identification of miRNAs in various biological samples, including serum, plasma, and whole blood, as well as in malignant tissues, has been conducted to elucidate their role in the progression of the disease, including but not limited to the following: cell proliferation, invasion, metastasis, and apoptosis. The presence of MPE as a consequence of LC has been regarded as a significant biological specimen in which the presence of miRNAs can be identified. However, the available research on this subject is limited. The present review focuses on the compilation of studies in which the presence of miRNAs in MPE from patients with LC. In eight studies, 17 miRNAs were described, including the following: miR-19a, miR-19b, miR-21, miR-22, miR-24, miR-25, miR-29c, miR-30d, miR-126, miR-134, miR-1-3p, miR-144-5p, miR-145, miR-150-5p, miR-182, miR-185, and miR-198. These miRNAs exhibited elevated AUC values, sensitivity, and specificity. Further studies are required to investigate MPE in a larger cohort of LC patients. As indicated by the various algorithms available for predicting gene regulation, there is a probability that thousands of genes are regulated by miRNAs. However, at present, only a few genes are directly regulated by certain miRNAs with LC. The following perspectives are taken into consideration in this study: 1. identification of a broad panel of miRNAs expressed in the PE of LC patients was conducted, with the subsequent establishment of possible associations with early prognostic factors or disease progression; 2. conduct studies with a large sample size were conducted on malignancies and benign lung diseases; 3. find molecular targets regulated by the aforementioned miRNAs that participate in the hallmarks of cancer, particularly invasion and metastasis, due to the diagnosis of the disease is in the late stages. 4. identify potential miRNAs that favor LC treatment, whether through expression or inhibition; and 5. determine whether miRNAs, in addition to other routine clinical biomarkers, can increase specificity and sensitivity, thereby allowing for an individualized therapeutic approach that reduces chemoresistance.
